# Incidental Gynecological Premalignant and Malignant Lesions in Patients Undergoing Hysterectomy for Benign Indications

**DOI:** 10.12669/pjms.42.4.13825

**Published:** 2026-04

**Authors:** Gokcen Ege, Hasan Volkan Ege, Huseyin Levent Keskin, Ayse Filiz Yavuz

**Affiliations:** 1 Gokcen Ege, MD, GE, Department of Gynecologic Oncology, Etlik City Hospital, Ankara, Turkey; 2 Hasan Volkan Ege, MD, HVE, Department of Gynecologic Oncology, Etlik City Hospital, Ankara, Turkey; 3 Huseyin Levent Keskin, MD, HLK Department of Obstetrics and Gynecology, Ufuk University Faculty of Medicine, Ankara, Turkey; 4 Ayse Filiz Avsar, MD, AFY Department of Obstetrics and Gynecology, Yildirim Beyazit University, Faculty of Medicine, Ankara, Turkey

**Keywords:** Hysterectomy, Incidental malignancy, Ovarian neoplasm, Premalignant lesions, Serous tubal intraepithelial carcinoma, Uterine sarcoma

## Abstract

**Objective:**

To determine the frequency and clinicopathological features of incidental premalignant and malignant gynecological lesions detected after hysterectomies performed for benign indications, and to identify associated risk factors.

**Methodology:**

This retrospective study reviewed 1,047 hysterectomies performed for benign conditions at a tertiary center in Ankara, Turkiye, over a ten years period (January 2006-December 2015). Demographic characteristics, preoperative assessments, surgical indications, and histopathological outcomes were analyzed. Incidental lesions were defined as pre-malignant or malignant pathologies identified postoperatively without prior clinical suspicion. Statistical analyses were performed using chi-square and t-tests.

**Results:**

Incidental pre-malignant or malignant lesions were identified in 6% (n=63) of cases, including cervical dysplasia/HSIL (0.9%), borderline ovarian tumors (1.1%), endometrial adenocarcinoma (0.5%), leiomyosarcoma (0.5%), and high-grade serous ovarian carcinoma (0.6%). Postmenopausal women had a significantly higher incidence than premenopausal women (9.2% vs. 3.8%, p<0.001). Patients with incidental findings were older than those with benign pathology (55.4 ± 11.2 vs. 52.7 ± 9.6 years, p=0.034). Larger myomas were associated with uterine sarcoma (172 ± 92 mm vs. 71 ± 39 mm, p<0.001). Ovarian malignancies were detected in 2.4% of cases without suspicious ultrasound features and in 14.8% of cases with ≥2 malignancy criteria (p=0.013). Serous tubal intraepithelial carcinoma (STIC) was identified in 0.28% (n=3).

**Conclusion:**

Incidental pre-malignant or malignant lesions were present in 6% of hysterectomies performed for benign indications. Age, menopausal status, myoma size, and suspicious imaging features were significant predictors. Preoperative evaluation may not completely exclude the possibility of occult pathology; therefore, this risk should be discussed during preoperative counseling.

## INTRODUCTION

In the United States, 5.38 hysterectomies are performed per 1000 woman-years, with 4.81 of them for benign indications. Uterine leiomyomas are the most common indication for hysterectomy, accounting for approximately 37% of cases. Other common indications include abnormal uterine bleeding (AUB) at 19%, pelvic organ prolapse (POP) at 13%, and endometriosis at 12%.^1^ In a study conducted in China, leiomyoma accounted for 39% of cases, POP for 24.7%, endometriosis/adenomyosis for 19.9%, and other benign pathologies such as AUB, endometrial polyps, and infections for 16.3%.^2^ Similarly, data from Pakistan have shown a comparable pattern, with fibroids being the leading indication for abdominal hysterectomy (40%) and dysfunctional uterine bleeding the second most common (29%), and with histopathological analysis occasionally identifying unexpected pathology such as undifferentiated uterine sarcoma.^3^

Unexpected malignancies occur in 2.7% of hysterectomies for benign reasons. Uterine sarcoma incidence is 0.22%, endometrial cancer 1.02%, ovarian-peritoneal-fallopian tube cancer 1.08%, and metastatic disease 0.2% in the most comprehensive study.^4^ In Turkey, the rate of unexpected gynecological malignancy is 1.23-0.58%, endometrial cancer at 0-0.31%, uterine sarcoma at 0.66-0.12%, ovarian cancer at 0.19-0.1%, fallopian tube cancer at 0-0.01%, and cervical cancer at 0.28-0.03.^5^,^6^ No data are available regarding premalignant diseases.

This study reviewed hysterectomy cases from the past decade in our clinic, focusing on discrepancies between preoperative and postoperative diagnoses and incidental malignancies. We hypothesized that a non-negligible proportion of hysterectomies performed for benign indications harbor unexpected pre-malignant or malignant lesions, particularly among older and postmenopausal women.

## METHODOLOGY

This retrospective study was conducted in the Department of Obstetrics and Gynecology at Ankara Ataturk Training and Research Hospital, a tertiary referral center in Ankara, Turkiye, and included women who underwent hysterectomy for benign indications between January 2006 to December 2015.

### Ethical approval:

It was obtained from the institutional ethics committee of Ankara Ataturk Training and Research Hospital on January 20, 2016 (Decision No: 16).

Patient information included age, gravidity, parity, clinical symptoms, a personal or family history of malignancy, and tumor markers if assessed. Preoperative pathology tests (Pap smear, endometrial sampling, cervical biopsy, paracentesis, etc.) and their results were documented. In assessing ovarian masses, findings of malignancy, such as multilocular cysts, solid components/papillary projections, bilaterality, and thick septa (>3 mm), were recorded. Intraperitoneal free fluid was classified as a serous effusion, mild ascites (<4 cm), and extensive ascites.

Indications for surgery, surgical techniques, and postoperative pathology results were documented. All surgical specimens were examined by experienced gynecologic pathologists according to the institutional pathology protocol. In cases where the fallopian tubes were removed, the fimbrial end of the tube was carefully examined. When suspicious areas were present, additional serial sectioning was performed. STIC diagnoses were established based on characteristic morphological features and, when required, immunohistochemical findings. During the early years of the study period, the SEE-FIM (Sectioning and Extensively Examining the FIMbria) protocol was not routinely implemented in our institution, as this technique had only recently been introduced into routine pathological practice.^7^

Pathology results were categorized into high-grade squamous intraepithelial lesion (HSIL), atypical endometrial hyperplasia (AEH), endometrial intraepithelial neoplasia (EIN), borderline ovarian tumor (BOT), serous tubal intraepithelial carcinoma (STIC) or primary fallopian tube cancer (PFTC), uterine sarcoma, endometrial cancer, cervical cancer, ovarian cancer, and metastatic cancers. Incidental malignancies were defined as those detected postoperatively without a preoperative cancer diagnosis or suspicion.

### Statistical analysis:

was conducted using IBM SPSS Statistics Version 21.0 software. Descriptive data were presented as mean ± standard deviation. Categorical data were compared between groups using the chi-square test, and continuous numerical data were compared using the t-test. A p-value of <0.05 was considered statistically significant.

## RESULTS

**Fig.1:**
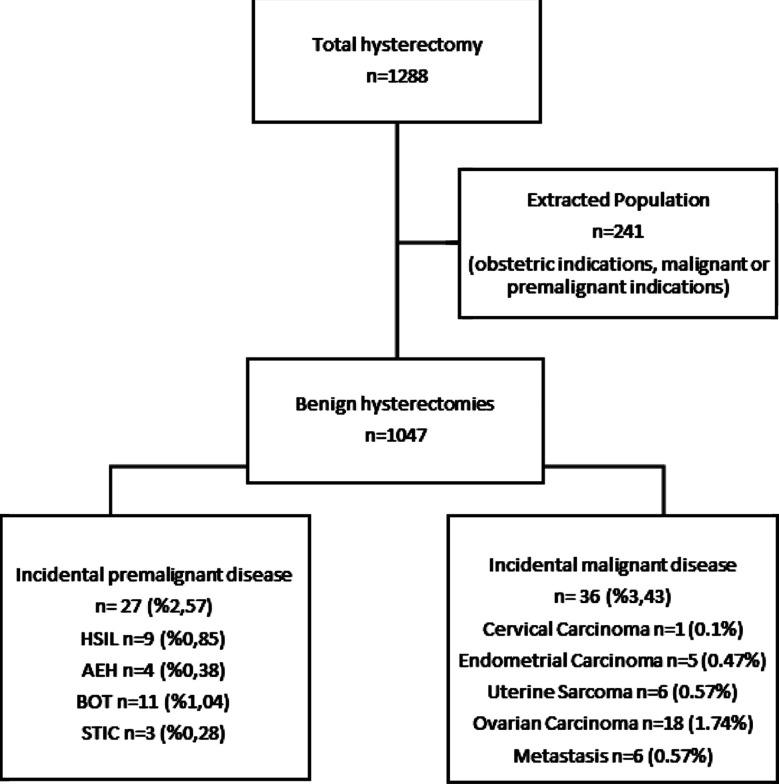
Flowchart illustrating inclusion, exclusion, and rates of incidental pre-malignant and malignant pathologies.

A total of 1047 patients were included (Fig.1). The mean age of the patients was 52.8 ± 9.7 years, with an average gravida of 4.5 ± 2.7 and parity of 3.3 ± 2.1. Of the 1047 patients, 59.7% were premenopausal, and 40.3% were postmenopausal (Table-I).

**Table-I:** Demographic Data of Patients.

Age (mean ± SD):	52.8 ± 9.7
Gravidity (mean ± SD):	4.5 ± 2.7
Parity (mean ± SD):	3.3 ± 2.1
Premenopausal (n; %):	625, 59.7%
Smokers (n; %):	147, 14%

The most common procedure was total abdominal hysterectomy with bilateral salpingo-oophorectomy (TAH+BSO) at 78.4%. This was followed by vaginal hysterectomy (VH) at 11.2% (n=117), TAH+unilateral salpingo-oophorectomy (TAH+USO) at 3.7% (n=39), TAH+bilateral salpingectomy at 3.3% (n=35), TAH at 2.4% (n=25), laparoscopic hysterectomy (LH) at 0.5% (n=5), and robotic hysterectomy (RH) at 0.5% (n=5). The most common indications were leiomyoma uteri (42%, n=440), adnexal mass (16.2%, n=170), abnormal uterine bleeding (13.8%, n=144), and pelvic organ prolapse (12.6%, n=132), as shown in Table-II.

**Table-II:** Operation Indications.

Indication	%	n
Myoma uteri	42	440
Complicated ovarian cyst	16.2	170
Abnormal uterine bleeding	13.8	144
Pelvic organ prolapse	12.6	132
Endometrial polyp	5.7	60
Simple ovarian cyst	5.5	58
Pelvic inflammatory disease - Tubo-ovarian abscess	2.8	29
Endometrioma	1	10
Torsion	0.3	3
Hematocolpos	0.1	1
Total	100	1047

It was found that 984 patients (94%) had benign results, while 63 patients (6%) had an incidental malignancy or premalignant lesions. Among these cases, there were 27 cases (2.57%) of premalignant lesions and 36 cases (3.43%) of malignancy (Fig.1). The pathology results of the 63 patients are shown in Table-III. The most commonly found incidental premalignant lesion was HSIL (n=9; 0.9%), while the most common malignancy was serous ovarian carcinoma (n=6; 0.6%).

**Table-III:** Incidental pre-/malign lesions detected in pathological evaluation.

	%	n
***Premalignant Lesions***		
Cervical Dysplasia - HSIL	0.87	9
AEH	0.38	4
Serous Borderline Ovarian Tumors	0.57	6
Mucinous Borderline Ovarian Tumors	0.47	5
STIC - PFTC	0.28	3
Total	2.57	27
***Malignant Lesions***		
***Cervical Cancers***		
Cervical Adenocarcinoma	0.1	1
Endometrial Cancers		
Endometrial Adenocarcinoma	0.47	5
***Uterine Sarcomas***		
Uterine leiomyosarcoma	0.47	5
Endometrial Stromal Sarcoma	0.1	1
***Ovarian Cancers - Epithelial Origin***		
High-Grade Serous Ovarian Tumors	0.57	6
High-Grade Mucinous Ovarian Tumors	0.1	1
Endometrioid-Type Ovarian Tumor	0.1	1
Clear Cell Ovarian Tumor	0.1	1
***Ovarian Cancers - Sex Cord-Stromal Origin***		
Granulosa-Theca Cell Tumor	0.47	5
Sertoli-Leydig Cell Tumor	0.1	1
Steroid Cell Tumor (SCT-NOS)	0.1	1
***Ovarian Cancers - Mesenchymal Origin***		
Fibrosarcoma	0.1	1
Carcinosarcoma	0.1	1
***Other***		
Lymphoma	0.1	1
Metastasis	0.47	5
Total	3.43	36
Overall Total	6	63

Premalignant or malignant pathology was significantly more common in postmenopausal patients (9.2%) compared to premenopausal patients (3.8%, p<0.001). Patients with incidental findings were older (mean age 55.4 years) than those with benign results (52.7 years, p=0.034). Tumor markers were higher in patients with malignancies: CA 125 (413 ± 2402 U/ml) vs. benign cases (35.9 ± 91 U/ml, p=0.001), CA 19-9 (71 ± 364 U/ml) vs. benign (19 ± 84 U/ml, p=0.019), and CEA (4.4 ± 11.1 ng/ml) vs. benign (1.8 ± 1.8 ng/ml, p<0.001).

### Cervical pathologies:

Among 819 Pap smears, 808 (98.7%) had benign results, 8 (1%) had ASC-US, 1 (0.1%) had AGUS, and 1 (0.1%) had benign endometrial cells detected. The rate of incidental premalignant or malignant pathologies was 5.4% (n=44), while in 228 cases with no smear taken or results unavailable, it was 8.3% (n=19) (p=0.099). This group includes lesions related to both cervical and other pathologies. When considering only pathologies related to the cervix, incidental cervical lesions were found in 0.8% (n=6) of the population with benign smear tests in the preoperative period and 1.9% (n=4) in the group without a smear or with unknown results (p=0.152).

### Endometrial pathologies:

Preoperative endometrial sampling was performed in 48.3% of cases. In those with benign findings, 4.5% had incidental pre-malignant and malignant lesions, compared to 7.2% in those without biopsy (p=0.077). Endometrial thickness (ET) was similar between patients with benign and malignant results (8.1 ± 6.7 mm vs. 8.0 ± 7.3 mm, p=0.943). However, patients with incidental endometrial lesions had significantly thicker preoperative ET (13 ± 9.4 mm, p=0.011).

### Uterine Sarcomas:

Uterine sarcomas, including leiomyosarcoma (n=5) and Mullerian adenosarcoma (n=1), were detected in 0.95% of patients with myomas. Larger myomas (172 ± 92 mm) were associated with sarcoma diagnoses (p<0.001). Among patients with a single mass, 4% were diagnosed with uterine sarcomas, while among cases with multiple masses, the number of patients diagnosed with sarcomas was 2 (0.5%) (p=0.043).

### Ovarian Pathologies:

Among the cases with ovarian cysts identified by ultrasonography (n=318), 85 (26.7%) showed no sonographic signs of malignancy. In 152 cases (47.8%), only one sign was present, while 61 cases (19.2%) had two signs, 16 cases (5%) had three signs, and four cases (1.3%) had all four signs together. The tumor markers of these cases were within the normal range, and according to the Risk of Malignancy Index (RMI), they were not considered high-risk cases.

Incidental ovarian malignancies were found in 2.4% of cases without preoperative signs of malignancy. The incidence increased with the number of sonographic signs: 7.9% with one sign and 14.8% with two or more (p=0.013). Twenty-six patients were diagnosed postoperatively with borderline or invasive ovarian malignancies, with similar CA 125 values compared to benign cases (76 ± 108 U/ml vs. 74 ± 795 U/ml, respectively) (p=0.990). However, CA 19-9 was significantly higher in malignant cases (111 ± 477 U/ml, p=0.001).

In 0.28% of the cases, primary fallopian tube cancer was detected. One patient was operated on due to myoma uteri and was incidentally found to have a focus of intraepithelial carcinoma in the tube. The second patient underwent surgery for a complex ovarian mass, and a diagnosis of high-grade serous carcinoma in the ovary, accompanied by a focus on serous tubal intraepithelial carcinoma (STIC), was made. The third patient had a history of breast cancer and tamoxifen use. She underwent surgery for endometrial pathology, and the pathology report revealed a primary fallopian tube carcinoma (PFTC) and a second focus of STIC. The characteristics of the patients are presented in Table-IV.

**Table-IV:** Characteristics of patients diagnosed with postoperative primary fallopian tube cancer.

Case	1	2	3
Age	55	60	53
GP	G5 P3	G5 P2	G0
Menopausal Status	Premenopausal	Postmenopausal	Postmenopausal
Symptoms	Menorrhagia	Pelvic pain	Asymptomatic
Preoperative Indication	Myoma Uteri	Complex Ovarian Mass	Endometrial Polyp and Tamoxifen use for breast cancer
Imaging Report	USG: Multiple myoma uteri, the largest measuring 78 mm in diameter. Normal ovaries	CT: Thick septated, contrast-enhancing semi-solid lesion extending from the pelvis to the umbilicus. 3 cm ascites in Douglas	USG: Endometrium thickness 22 mm, normal ovaries and Douglas
Preoperative Pathology Results	Pap-smear: Benign EMB: Benign	Pap-smear: Benign	EMB: Endometrial Polyp
CA 125 (U/ml)	5.2	478	18.1
CA 19-9 (U/ml)	7.3	21	2.8
CA 15-3 (U/ml)	15.7	71.7	12.7
CEA (ng/ml)	0.8	1.4	1.1
Operation	TAH + BSO	TAH + BSO + BPPLND	TAH + BSO
Postoperative Pathology Results	High-grade Serous Carcinoma, left tubal fimbrial end (tumor size 5x3 mm)	High-grade Serous Carcinoma (left ovarian mass), High Grade Serous Tubal Intraepithelial Carcinoma (STIC) in fimbria	High-grade Serous Carcinoma, right tubal fimbrial end (tumor size 5 mm), Serous Tubal Intraepithelial Carcinoma (STIC) in right tuba uterina, second lesion

BPPLND: Bilateral pelvic and para-aortic lymph node dissection; CT: Computed Tomography; EMB: Endometrial biopsy; TAH+BSO: Total Abdominal Hysterectomy + Bilateral Salpingo-Oophorectomy; USG: Ultrasonography.

## DISCUSSION

In this 10 years cohort of 1,047 women who underwent hysterectomy for benign indications, we found incidental pre-malignant or malignant gynecological lesions in 6% of cases, including 2.6% pre-malignant and 3.4% malignant pathologies. High-grade squamous intraepithelial lesion (HSIL) was the most frequent pre-malignant lesion, whereas serous ovarian carcinoma was the most common invasive malignancy. Incidental pathology was significantly more frequent in postmenopausal women than in premenopausal women and was associated with older age, larger myomas, elevated tumor markers, and suspicious adnexal ultrasound findings. These results confirm that a non-negligible proportion of women undergoing hysterectomy for apparently benign disease harbor occult gynecologic pathology.

The rate of unexpectedly diagnosed gynecologic malignancies in women undergoing hysterectomy for benign reasons has been reported as approximately 2.7% in the most extensive series, including 7,499 cases, with incidences of 0.22% for uterine sarcoma, 1.02% for endometrial cancer, 1.08% for ovarian-peritoneal-fallopian tube cancer, and 0.2% for metastatic disease.^2^,^4^,^8^ Turkish data suggest an unexpected gynecologic malignancy rate ranging from 0.58% to 1.23%, with endometrial cancer at up to 0.31%, uterine sarcoma at 0.12-0.28%, ovarian cancer at 0.10-0.19%, fallopian tube cancer at 0.01%, and cervical cancer at 0.03-0.28%.^5^,^6^ In our cohort, the overall incidence of incidental pre-malignant or malignant lesions (6%) was higher than previously reported, which may reflect both our center’s comprehensive histopathological review policy and the broader inclusion of pre-malignant lesions such as HSIL, EIN/AEH, borderline ovarian tumors, and STIC. Our findings also complement data from the Pakistani series, in which leiomyoma was the leading indication for hysterectomy, and histopathological evaluation occasionally revealed unexpected pathology, including rare malignancies.^3^

Another factor that may explain the relatively higher rate of incidental malignancy in our cohort is that our institution functions as a tertiary referral center. Patients with complex adnexal masses, large uterine tumors, or inconclusive preoperative evaluations are frequently referred to our center from secondary hospitals. This referral pattern may lead to a higher concentration of patients with potentially occult malignancies compared with population-based hysterectomy series. Therefore, the 6% rate observed in our study may partially reflect referral bias rather than the true incidence in the general population.

### Age and Menopausal Status as Predictors of Malignancy:

A significant finding in our study is the relationship between patient age and the likelihood of detecting premalignant or malignant lesions. The mean age of patients with incidental pathologies was significantly higher than those with benign results (55.4 ± 11.2 vs. 52.7 ± 9.6, respectively, p=0.034). This is in line with numerous studies showing that the risk of malignancy increases with age, especially in postmenopausal women.^9^,^10^ Our data showed that postmenopausal women had a significantly higher rate of premalignant or malignant findings compared to premenopausal women (9.2% vs. 3.8%, p<0.001). These findings suggest that clinicians should maintain a high index of suspicion for malignancy when planning hysterectomies in older or postmenopausal women, even when preoperative evaluations suggest benign conditions.

### Significance of Tumor Markers and Imaging Findings:

Our study also underscores the importance of preoperative tumor markers and imaging in identifying patients at increased risk for incidental malignancies. Elevated CA 125 levels are strongly associated with malignant outcomes^11^,^12^. The average CA 125 level in cases with premalignant or malignant pathology was 413 ± 2402 U/ml, significantly higher than in benign cases (35.9 ± 91 U/ml, p=0.001). Preoperative imaging findings, especially the presence of ascites or complex ovarian masses, were also strongly associated with incidental malignant pathologies.

### Role of Preoperative Screening for Endometrial and Cervical Lesions:

Preoperative endometrial sampling and Pap smears play an essential role in screening for premalignant or malignant lesions. In our study, 48.3% of patients underwent preoperative endometrial sampling, with 4.5% of these patients being diagnosed with incidental pre-malignant or malignant lesions postoperatively. Endometrial sampling was not routinely performed in all patients because a substantial proportion underwent hysterectomy for indications such as pelvic organ prolapse or adnexal pathology without abnormal uterine bleeding or suspicious endometrial findings, according to institutional practice during the study period. The rate was higher in those without biopsy (7.2%), although this difference was not statistically significant (p=0.077). These findings suggest that endometrial sampling may not capture all premalignant or malignant lesions preoperatively, especially in asymptomatic patients.^13^ Similarly, our findings indicate that while a benign Pap smear was associated with a lower rate of incidental cervical pathology (0.8% vs. 1.9%, p=0.152), there remains a possibility of missed diagnoses. Given that only 48% of patients in our cohort underwent endometrial biopsy and approximately 98.7% had benign Pap smears, the possibility of incidental malignancies is not negligible. This suggests that while these preoperative tests are helpful, they may not entirely eliminate the risk of an incidental diagnosis.

### Unexpected Uterine Sarcomas:

The detection of uterine sarcomas (0.57%) in our study aligns with the literature. Incidence studies on this topic have reported a uterine leiomyosarcoma detection rate ranging from 0.22% to 0.49%, with the most extensive Desai et al. study finding rates of 0.22% for sarcoma and 0.15% for leiomyosarcoma.^8^ Mahnert et al. and Theben et al. found rates of 0.22% and 0.18%, respectively.^4^,^14^ Uterine sarcomas are notoriously difficult to diagnose preoperatively due to their nonspecific clinical and imaging features.^15^,^16^ In our cohort, patients with larger myomas were more likely to have sarcomas (172 ± 92 mm, p<0.001), which suggests that large, rapidly growing fibroids, particularly in postmenopausal women, warrant a high level of suspicion for sarcoma.

### Unexpected Ovarian and Tubal Malignancies:

In recent years, serous tubal intraepithelial carcinoma (STIC) has been considered a precursor lesion for high-grade serous carcinomas, the most common epithelial ovarian cancer.^17^ This pathology is often detected incidentally in the fimbrial end of the fallopian tube, initially in BRCA mutation carriers but later in non-high-risk patients.^18^ In our cases, STIC was found in 0.28% of patients, which aligns with the rates of less than 0.5% reported in other studies.^19^ None of the patients had symptoms of abundant watery vaginal discharge. STIC was found at the fimbrial end of the tube or coexisted with a high-grade serous carcinoma in the ovary. While it may not be cost-effective to create a screening test for a rare disease that remains asymptomatic, recent studies suggest that performing total salpingectomy in patients who will undergo surgery for other reasons can reduce the incidence of serous ovarian cancer, compared to the group where the tubes are left, or tubal ligation is performed.^20^ In contrast to previous Turkish reports, where rates of incidental malignancy ranged between 0.5-1.2%, our findings suggest a broader spectrum of occult pathology.

In recent years, opportunistic salpingectomy during hysterectomy for benign indications has gained increasing acceptance as a preventive strategy for epithelial ovarian cancer. Recent international guidelines and large population-based studies published between 2023 and 2024 support the removal of fallopian tubes during benign gynecologic surgery to reduce the future risk of high-grade serous ovarian carcinoma. Our observation of incidental STIC lesions further supports the concept that the fallopian tube may serve as the origin of many serous carcinomas and highlights the potential preventive value of opportunistic salpingectomy.^20^-^24^

### Clinical implications:

The findings of this study have important clinical implications for the management of patients undergoing hysterectomy for benign indications. First, careful preoperative evaluation, including the use of tumor markers, imaging, and endometrial sampling, should be considered in all patients, particularly those who are older, postmenopausal, or have concerning symptoms. Second, clinicians should maintain a high level of suspicion for malignancy, even in the absence of typical clinical or radiological signs. In particular, our results suggest that the presence of elevated tumor markers, large myomas, or complex adnexal masses should prompt further investigation to rule out malignancy before proceeding with surgery.

### What this study adds to the literature?

This study adds several novel aspects to the existing literature on incidental pathology at hysterectomy. First, unlike many previous reports focusing exclusively on uterine malignancies, we systematically evaluated a broad spectrum of pre-malignant and malignant lesions, including cervical HSIL, AEH/EIN, borderline ovarian tumors, STIC and primary fallopian tube carcinoma within the same cohort. Second, we showed that even in a setting where Pap smears and opportunistic endometrial sampling are frequently performed, a clinically meaningful proportion of cervical and endometrial lesions remain undetected preoperatively. Third, our detailed analysis of tumor markers, uterine size, and adnexal ultrasound characteristics provides practical information that can help clinicians stratify the risk of occult pathology when counseling women undergoing hysterectomy for benign indications.

### Strengths and Areas for further research:

Strengths of this study include the relatively large sample size, the 10 years observation period, and the uniform surgical and pathological protocols of a single tertiary center. However, further prospective, multicenter studies with standardized preoperative imaging and systematic long-term follow-up are needed to validate these findings. Future research should also assess the cost-effectiveness of more intensive preoperative evaluation and the potential impact of broader use of opportunistic salpingectomy and bilateral salpingo-oophorectomy in selected risk groups.

### Limitations:

The main limitation of this study is that it was conducted at a tertiary center, and patients referred from other centers were operated on. Therefore, it may not accurately reflect the incidence of the entire population. Additionally, it was a retrospective study, and only existing data could be analyzed. Furthermore, follow-up data regarding patient outcomes after incidental findings were unavailable, limiting prognostic interpretation.

## CONCLUSION

Our study provides valuable insights into the prevalence of incidental premalignant and malignant lesions in women undergoing hysterectomy for benign indications. While the overall rate of unexpected malignancies is relatively low, certain risk factors, such as advanced age, postmenopausal status, and elevated tumor markers, can significantly increase the likelihood of detecting malignancies. However, preoperative assessment cannot entirely exclude the risk of occult malignancy. Therefore, patients should be informed about the small but significant risk of incidental pre-malignant and malignant lesions during preoperative counseling, especially those who are postmenopausal or present with large or complex masses. Future prospective studies are warranted to validate these findings and to establish improved preoperative risk assessment strategies.
